# Effects of tafamidis on serial [^99m^Tc]Tc-DPD scintigraphy in transthyretin amyloid cardiomyopathy

**DOI:** 10.1007/s00259-025-07092-7

**Published:** 2025-02-06

**Authors:** Maria Ungericht, Thomas Schuetz, Moritz Messner, Christian Puelacher, Simon Staggl, Marc-Michael Zaruba, Alexander Stephan Kroiss, Axel Bauer, Gerhard Poelzl

**Affiliations:** 1 Department of Internal Medicine III, Cardiology and Angiology, Medical University of Innsbruck, Anichstraße 35, 6020 Innsbruck, Austria; 2 Department of Nuclear Medicine, Medical University of Innsbruck, Innsbruck, Austria; 3 Department of Nuclear Medicine, General Hospital Barmherzige Schwestern, Ried Im Innkreis, Austria

**Keywords:** Amyloidosis, Transthyretin, [^99m^Tc]Tc-DPD scintigraphy, Tafamidis, Monitoring

## Abstract

**Purpose:**

The relevance of repetitive [^99m^Tc]Tc-DPD scintigraphy in wild-type transthyretin amyloid cardiomyopathy (ATTRwt-CM) remains unclear. We investigated the impact of tafamidis on cardiac [^99m^Tc]Tc-DPD uptake, clinical, and laboratory markers at 6 and 12 months, and correlated 12 months [^99m^Tc]Tc-DPD uptake regression with survival.

**Methods:**

This single-center study enrolled 39 ATTRwt-CM patients. Upon treatment initiation with tafamidis, patients underwent follow-up [^99m^Tc]Tc-DPD scintigraphy, and clinical and laboratory evaluations at 6 months (*n* = 6) and 12 months (*n* = 13), or both (*n* = 20).

**Results:**

Tafamidis resulted in a significant decline in Perugini score (6 months *p* = 0.008, 12 months *p* < 0.001), and (semi-)quantitative [^99m^Tc]Tc-DPD uptake (total cardiac uptake: baseline 816 [522–933] cps, vs. 6 months 634 [502–734] cps, *p* = 0.003, vs. 12 months 523 [108–754] cps, *p* = 0.001). Clinical and laboratory improvements were observed (NYHA: 6 months *p* = 0.007, 12 months *p* = 0.033; NT-proBNP: baseline 2586 [1271–5561] ng/L, vs. 6 months 2526 [1109–4786] ng/L, *p* = 0.016, vs. 12 months 2340 [1411–4749] ng/L, *p* = 0.012). In Kaplan–Meier analysis, a decrease in right ventricular [^99m^Tc]Tc-DPD tracer uptake equal to or greater than the median value at 12 months (-30%) was associated with improved survival (log-rank *p* = 0.021).

**Conclusions:**

Tafamidis in ATTRwt-CM resulted in significant reductions of cardiac [^99m^Tc]Tc-DPD uptake, NYHA class, and cardiac biomarkers at 6 and 12 months. Regression of right ventricular [^99m^Tc]Tc-DPD uptake at 12 months was associated with improved survival.

**Supplementary Information:**

The online version contains supplementary material available at 10.1007/s00259-025-07092-7.

## Introduction

Transthyretin amyloid cardiomyopathy (ATTR-CM) is a debilitating disease caused by the dissociation of the transthyretin tetramer protein into monomers, which form insoluble amyloid fibrils and accumulate within the myocardial interstitial tissue. This either can arise from a mutation in the *TTR* gene (variant ATTR-CM, ATTRv-CM) or be related to aging (wild-type ATTR-CM, ATTRwt-CM). If left untreated, the disease is associated with a rapid decline in functional capacity, diminished quality of life, and poor prognosis [1].

With the advent of novel disease-modifying therapies, morbidity and mortality can be reduced [2, 3]. Tafamidis was the first transthyretin tetramer stabilizer to be approved for the treatment of ATTR-CM and has been incorporated into international guidelines [4, 5]. The corresponding landmark trial demonstrated its efficacy in decreasing all-cause mortality and cardiovascular-related hospitalizations, while reducing the decline in functional capacity and quality of life [2, 3]. Studies utilizing transthoracic echocardiography (TTE) and cardiac magnetic resonance imaging (CMR) have revealed that tafamidis delays phenotypical ATTR-CM progression. These findings have been correlated with clinical benefits when compared to untreated ATTR-CM patients [6, 7].

Precise assessment of cardiac amyloid load is of utmost importance for therapy monitoring recognizing that promising alternative therapies will be available in the near future [8]. Disease monitoring by imaging is primarily performed by TTE and CMR. However, while TTE and CMR can be used for the assessment of cardiac structure and function, bone scintigraphy provides a window into the underlying pathophysiological process.

Technetium-99 m [^99m^Tc]-labeled 3,3-diphosphono-1,2-propanodicarboxylic-acid (DPD) scintigraphy plays a pivotal role in diagnosing ATTR-CM in the setting of strong cardiac tracer accumulation (Perugini score ≥ 2), provided systemic monoclonal proteins are excluded [9]. [^99m^Tc]Tc-DPD scintigraphy involves planar imaging followed by single-photon emission computed tomography/computed tomography imaging (SPECT/CT). SPECT/CT is crucial for distinguishing cardiac tracer uptake from regional rib tracer uptake, diffuse skeletal muscle uptake, and tracer retention in the left ventricular blood pool [10–12]. It also provides a decision-making aid in the event of borderline tracer uptake on planar scintigraphy (Perugini < 2). Semiquantitative scintigraphic analysis holds prognostic value, with higher ratios indicating more intense cardiac tracer uptake, being associated with worse survival rates [13, 14].

The efficacy of [^99m^Tc]-labeled bone scintigraphy as a disease monitoring tool is still under investigation. Previous studies showed significant correlations between cardiac tracer uptake and histological ATTR burden in endomyocardial biopsies [15, 16]. These results suggest that myocardial tracer uptake truly reflects the invasive amyloid burden, which is why serial bone scintigraphy may play a role in treatment monitoring. Real-world studies have observed reductions in cardiac [^99m^Tc]Tc-DPD tracer uptake during follow-up in ATTR-CM patients receiving amyloid-specific therapy, potentially indicating amyloid regression [17–19]. However, whether scintigraphic tracer regression during follow-up is associated with clinical and laboratory benefits, and improved outcome is still unclear.

The aims of this study were to investigate:

(i) The effects of tafamidis on cardiac [^99m^Tc]Tc-DPD uptake in patients with ATTR-CM who underwent serial scintigraphic imaging at 6 or 12 months, or both.
(ii) The longitudinal changes in functional capacity and cardiac biomarkers.
(iii) The association between changes in cardiac tracer uptake and outcome.

## Methods

### Study design

This single-center registry study was conducted at the amyloidosis referral center of the Medical University of Innsbruck, Department of Cardiology and Angiology. Patients diagnosed with ATTR-CM were recruited between June 2018 and May 2023 (retrospective patient inclusion: June 2018-May 2021, prospective patient inclusion: June 2021-May 2023). Diagnosis of ATTR-CM relied on the presence of intense myocardial tracer accumulation on [^99m^Tc]Tc-DPD scintigraphy (Perugini score ≥ 2) and the absence of monoclonal proteins. In cases of discordant imaging findings or the presence of monoclonal proteins, endomyocardial biopsy was performed. Genotyping was performed to differentiate between ATTRwt-CM and ATTRv-CM. ATTR-CM patients with available [^99m^Tc]Tc-DPD planar scintigraphy and SPECT/CT were included, while those with ATTRv-CM were excluded. The study population underwent comprehensive baseline diagnostic work-up, including TTE, assessment of clinical status, functional capacity and measurement of cardiac biomarkers. Upon confirmation of ATTR-CM diagnosis, tafamidis 61 mg once daily was started. Treatment costs were either covered by a “compassionate use program” or – after approval of tafamidis in Austria – by the health insurance company. Follow-up visits were performed at 6 or 12 months, or both, as determined by the treating physician. The study received approval from the local ethics committee (1140/2021) and was conducted in accordance with the Declaration of Helsinki. For the study report we adhered to the STROBE guidelines.

### [^99m^Tc]Tc-DPD scintigraphy (planar, SPECT/CT)

[^99m^Tc]Tc-DPD scintigraphy was performed using a Philips Brightview XCT gamma camera applying an activity of 550 MBq (baseline: 39 patients, 6 months: 26 patients, 12 months: 33 patients). Planar whole-body imaging and SPECT/CT scans of the chest were obtained 3 h following [^99m^Tc]Tc-DPD tracer application. Image analysis was conducted by an experienced specialist in nuclear medicine. Cardiac [^99m^Tc]Tc-DPD uptake was visually assessed on planar whole-body scans using the Perugini scoring system where a score of 0 indicated no cardiac uptake with normal bone uptake, a score of 1 signified mild cardiac uptake lower than bone uptake, a score of 2 described moderate cardiac uptake with reduced bone uptake, and a score of 3 represented strong cardiac uptake with mild or absent bone uptake [20]. Quantitative analysis involved measuring [^99m^Tc]Tc-DPD uptake in different regions of the left ventricle (LV) (septal, lateral, anterior, posterior) and the right ventricle (RV). In addition, total cardiac tracer uptake was measured including left and right ventricular tracer uptake. Semiquantitative assessment of tracer uptake in the heart and a corresponding reference structure was accomplished by drawing two-dimensional regions of interest (ROI). Mean counts of emitted radioactivity per pixel per second (cps) were recorded for each ROI. A ROI was placed over the heart and then copied onto the mediastinum (above the cardiac ROI). The heart-to-mediastinum ratio (H/M) was calculated as the mean cps in the cardiac ROI divided by the mean cps in the mediastinum ROI. On planar scans, the heart-to-corpus-sterni ratio (H/CS) was calculated as the mean cps in the heart divided by the mean cps in the corpus-sterni.

### Clinical, laboratory, and echocardiographic assessments

Patients underwent detailed baseline and follow-up assessments. These were carried out at the Department of Cardiology and Angiology at the Medical University of Innsbruck, which runs a special clinic for rare myocardial diseases and acts as a reference center for amyloidosis. For retrospective patient inclusion from June 2018 to May 2021, clinical, laboratory and TTE assessments were retrieved from the digital medical record system and transferred to SPSS for subsequent statistical analysis. From June 2021 to May 2023, patients were enrolled prospectively.

Baseline and follow-up investigations included clinical assessments using the New York Heart Association (NYHA) functional class, and appraisal of functional capacity measured by the distance in the 6-min walk test (6MWT). Laboratory testing included measurements of N-terminal prohormone of brain natriuretic peptide (NT-proBNP), high sensitivity cardiac troponin T (hs-cTnT), and estimated glomerular filtration rate (eGFR).

Additionally, patients underwent TTE, which was performed using a Philips EPIQ CVx machine. From this, cardiac structure and function was documented by obtaining measurements (acquired with ImageArena software) of LV end-diastolic volume, LV ejection fraction (LVEF), interventricular septal thickness (IVS), LV mass, right ventricular systolic function and pulmonary artery pressure. Speckle tracking echocardiography of the LV also enabled analysis of global longitudinal strain (GLS).

### Outcome assessment

Our prognostic endpoint was all-cause death, which was correlated with the change in [^99m^Tc]Tc-DPD uptake at 12 months compared to baseline (in the LV and RV). Patients were followed up from the time of diagnosis until November 2, 2023, or until the occurrence of death, whichever occurred first. Death events were obtained either from our medical records or (if not available) from the official death register.

### Statistical analysis

Categorical variables were expressed as frequencies (percentages), while continuous variables were presented as mean ± standard deviation (SD) or median [interquartile range (IQR)]. Wilcoxon signed-rank test was used to test paired, not normally distributed data, and the paired *t*-test was used to analyze paired, normally distributed data. The distribution of data was assessed using the Kolmogorov–Smirnov test. The prognostic significance of [^99m^Tc]Tc-DPD uptake regression was calculated with both Kaplan–Meier analysis using the log-rank test (univariable analysis) and univariable and multivariable Cox proportional hazard regression analysis (multivariable analysis was adjusted for NYHA functional class and Mayo score [including NT-proBNP and hs-cTnT]) (time interval was calculated from; (1) date of [^99m^Tc]Tc-DPD scintigraphy at 12 months, and (2) date of death/November 2, 2023). A p-value < 0.05 was considered statistically significant. Statistical analysis was performed using IBM SPSS Statistics (version 29, IBM Corporation, Armonk, NY, USA). Figures were created using Prism 10 (GraphPad Software La Jolla CA, US).

## Results

### Baseline characteristics

Between June 2018 and May 2023, 39 patients (26% women) with ATTR-CM and available baseline results were included in the study. *TTR* genotyping was available for all patients, with only those diagnosed with ATTRwt-CM being considered for further analyses. Table 1 outlines the baseline characteristics of the study population.

**Table 1:** Baseline and follow-up parameters of ATTRwt-CM patients

Clinical parameters	Baseline (*n* = 39)	6-months-follow-up (*n* = 26)	*p*-value	12-months-follow-up (*n* = 33)	*p*-value
NYHA Class			**0.007**		**0.033**
Class I	2 (5%)	3 (12%)		6 (18%)	
Class II	23 (59%)	18 (69%)		16 (49%)	
Class III	14 (36%)	5 (19%)		11 (33%)	
6-min walk distance, meters	347 ± 115	356 ± 107	0.281	374 ± 136	**0.020**
Laboratory					
eGFR, mL/min/1.73m^2^	59 [43–60]	59 [43–60]	0.423	60 [41–60]	0.076
NT-proBNP, ng/L	2586 [1271–5561]	2526 [1109–4786]	**0.016**	2340 [1411–4749]	**0.012**
Hs-cTnT, ng/L	49.1 [29.4–70.8]	43.6 [27.8–69.5]	0.087	39.9 [27.9–70.6]	**0.007**
Echocardiography					
LVEDV, mL	94.7 ± 44.4	96.8 ± 34.9	0.645	103.1 ± 40.8	0.123
LVEF, %	53.9 ± 9.3	54.1 ± 11.5	0.203	54.7 ± 9.3	0.980
IVS, mm	17.7 ± 3.8	18.3 ± 3.6	0.559	17.5 ± 2.1	0.199
LV mass, g/m^2^	181 [143–211]	186 [121–220]	0.594	166 [153–188]	0.687
TAPSE, mm	17.3 ± 4.2	20.0 ± 3.2	0.715	20.1 ± 6.5	0.755
sPAP, mmHg	42 ± 13.6	41.8 ± 9.7	0.535	44.6 ± 12.8	0.969
GLS, -%	11.1 ± 4.5	9.8 ± 5.1	0.511	11.2 ± 3.8	0.620
[^99m^Tc]Tc-DPD scintigraphy					
*Planar scintigraphy*					
Perugini score			**0.008**		** < 0.001**
1	2 (5%)	1 (4%)		6 (18%)	
2	10 (26%)	14 (54%)		16 (49%)	
3	27 (69%)	11 (42%)		11 (33%)	
H/CS	3.2 [2.4–4.6]	2.4 [1.8–3.4]	** < 0.001**	1.7 [1.4–2.5]	** < 0.001**
*SPECT/CT analysis*					
Total cardiac uptake, cps	816 [522–933]	634 [502–734]	**0.003**	523 [108–754]	**0.001**
Left ventricle, cps	912 [582–1052]	686 [503–812]	**0.001**	584 [79–804]	**0.002**
Septal region, cps	1036 [518–1216]	816 [645–950]	**0.004**	697 [98–938]	**0.006**
Lateral region, cps	781 [403–970]	556 [385–677]	**0.004**	522 [72–729]	**0.003**
Anterior region, cps	757 [574–1032]	663 [408–742]	**0.003**	500 [75–766]	**0.002**
Posterior region, cps	913 [658–1128]	683 [486–901]	**0.013**	673 [76–836]	**0.013**
Right ventricle, cps	439 [180–597]	413 [224–499]	0.053	357 [62–434]	**0.012**
H/M	9.8 [7.5–11.7]	6.3 [5.4–10.4]	**0.048**	5.5 [4.5–7.0]	** < 0.001**

Values are n (%), mean ± SD, or median [IQR]

*p*-values denote the comparison between baseline and 6-/12-months-follow-up timepoint

[^99m^Tc]Tc-DPD: Technetium-99 m-labeled-3,3-diphosphono-1,2-propanodicarboxylic-acid; ATTRwt-CM: wild-type transthyretin amyloid cardiomyopathy; cps: counts of emitted radioactivity per pixel per second; eGFR: estimated glomerular filtration rate; GLS: global longitudinal strain; H/M: heart-to-mediastinum ratio; H/CS: heart-to-corpus-sterni ratio; hs-cTnT: high sensitivity cardiac troponin T; IVS: interventricular septal thickness; LV mass: left ventricular mass; LVEDV: left ventricular end-diastolic volume; LVEF: left ventricular ejection fraction; NT-proBNP: N-terminal prohormone of brain natriuretic peptide; NYHA: New York Heart Association functional class; PAP: pulmonary artery pressure; SPECT/CT: Single-photon emission computed tomography/computed tomography imaging; TAPSE: tricuspid annular plane systolic excursion

### Clinical parameters

The average age of the study cohort was 77 ± 5 years. Most patients were in NYHA functional class II (59%), with elevated levels of NT-proBNP (2586 [1271–5561] ng/L), and hs-cTnT (49.1 [29.4–70.8] ng/L). The mean distance covered in the 6MWT was 347 ± 115 m.

### Imaging parameters

Visual scoring of cardiac [^99m^Tc]Tc-DPD uptake was performed on planar imaging, with 2 (5%) patients being Perugini score 1, 10 (26%) score 2, and 27 (69%) score 3. The presence of intense cardiac tracer uptake was confirmed on quantitative and semiquantitative analyses using SPECT/CT (Table 1).

Echocardiography revealed preserved LVEF (53.9 ± 9.3%), along with increased IVS (17.7 ± 3.8 mm). Speckle-tracking echocardiography indicated impaired GLS (−11.1 ± 4.5%) and showed an apical sparing pattern among the majority of the study participants.

### Follow-up evaluation

After starting treatment with tafamidis 61 mg once daily, a follow-up examination was carried out in 6 patients after 6 [6-7] months, and in 13 patients after 12 [11-16] months, and 20 patients were followed up both after 6 and 12 months. Follow-up findings and comparison analyses are presented in Table 1.

### Clinical parameters and correlations

**Fig. 1:**
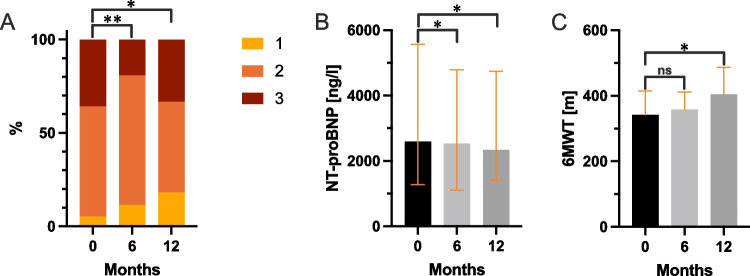
Changes in clinical and laboratory parameters (Baseline vs. 6 months vs. 12 months): (**A**) Changes in NYHA functional class at 6 (*p* = 0.007) and 12 months (*p* = 0.033). (**B**) Decline in NT-proBNP levels at 6 (*p* = 0.016) and 12 months (*p* = 0.012). (**C**) Improvements in 6MWT at 6 (*p* = 0.281) and 12 months (*p* = 0.020). (ns: not significant, *: *p* < 0.05, **: *p* < 0.01, ***: *p* < 0.001)

At 6 and 12 months, there were significant improvements in NYHA functional class (*p* = 0.007 and *p* = 0.033, respectively) (Fig. 1A). These findings were mirrored by a decrease in NT-proBNP (baseline: 2586 [1271–5561] ng/L, 6 months: 2526 [1109–4786] ng/L, *p* = 0.016, 12 months: 2340 [1411–4749] ng/L, *p* = 0.012) (Fig. 1B) and hs-cTnT (baseline: 49.1 [29.4–70.8] ng/L, 6 months: 43.6 [27.8–69.5] ng/L, *p* = 0.087, 12 months: 39.9 [27.9–70.6] ng/L, *p* = 0.007). Improvements were also seen in the 6MWT performance at 12 months (baseline: 347 ± 115 m, 6 months: 356 ± 107 m, *p* = 0.281, 12 months: 374 ± 136 m, *p* = 0.020) (Fig. 1C). eGFR remained stable over the 12 months.

### Imaging parameters and correlations

**Fig. 2:**
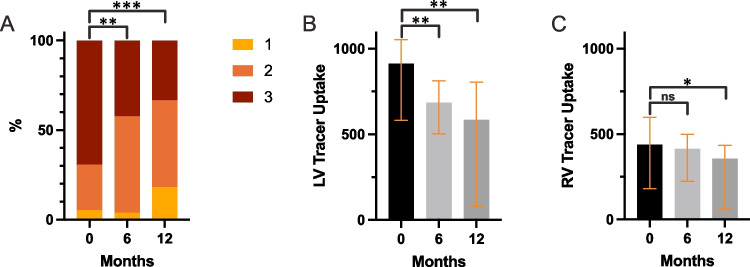
Changes in [^99m^Tc]Tc-DPD uptake (Baseline vs. 6 months vs. 12 months): (**A**) Changes in Perugini score at 6 (*p* = 0.008) and 12 months (*p* < 0.001) (planar). (**B**) Quantitative [^99m^Tc]Tc-DPD tracer regression in the LV (6 months: *p* = 0.001, 12 months: *p* = 0.002) and the (**C**) RV (6 months: *p* = 0.053, 12 months: 0.012) at 6 and 12 months (SPECT/CT). (ns: not significant, *: *p* < 0.05, **: *p* < 0.01, ***: *p* < 0.001)

Following treatment with tafamidis, serial [^99m^Tc]Tc-DPD planar imaging showed a consistent decline in Perugini score (Fig. 2A). At 6 months (*n* = 26), 19 patients showed unchanged Perugini score, while 7 patients improved by 1 grade (*p* = 0.008). At 12 months (*n* = 33), 19 patients displayed no change in Perugini score, 13 experienced an improvement by 1 grade, and 1 patient improved from Perugini score 3 to 1 (*p* < 0.001). Quantitative [^99m^Tc]Tc-DPD SPECT/CT analysis at 6 months revealed notable tracer regression in the LV (*p* = 0.001) (Fig. 2B), with a trend toward tracer regression in the RV (*p* = 0.053) (Fig. 2C). When applying semiquantitative analysis, significant reductions in H/M (*p* = 0.048) and H/CS (*p* < 0.001) were demonstrated at 6 months. At 12 months, substantial tracer regression was observed in the LV (*p* = 0.002) (Fig. 2B), along with evident decreases in RV tracer uptake (*p* = 0.012) (Fig. 2C). These findings were supported by a decline in semiquantitative analysis, with stark reductions in H/M (*p* < 0.001) and H/CS (*p* < 0.001) (Table 1).

Of the 20 ATTR patients undergoing follow-up bone scan at both 6 and 12 months, 12 patients showed stronger tracer regression between baseline and 6 months and milder tracer regression between 6 and 12 months. The remaining 8 patients had stronger tracer regression between 6 and 12 months and milder tracer regression between baseline and 6 months.

**Fig. 3:**
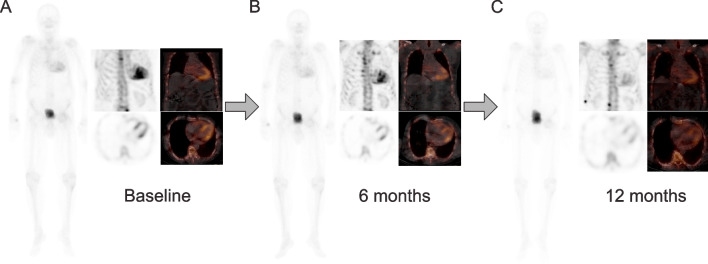
[^99m^Tc]Tc-DPD planar scintigraphy and SPECT/CT of an ATTRwt-CM patient treated with tafamidis: (**A**) Baseline vs. (**B**) 6 months vs. (**C**) 12 months

Figure 3 shows [^99m^Tc]Tc-DPD uptake regression on planar scintigraphy and SPECT/CT at 6 and 12 months in an ATTRwt-CM patient treated with tafamidis.

No significant differences were found in the follow-up echocardiographic examinations compared to the initial examinations (p > 0.05 for all comparisons). However, numeric improvements and stable findings were consistently recorded.

### Prognostic endpoint

The mean follow-up duration was 34 ± 13 months from the time of treatment initiation. No patient was lost to follow-up. In the overall study cohort, 11 patients had died. Among these, 10 patients had available 12 months follow-up [^99m^Tc]Tc-DPD scintigraphy.

**Fig. 4:**
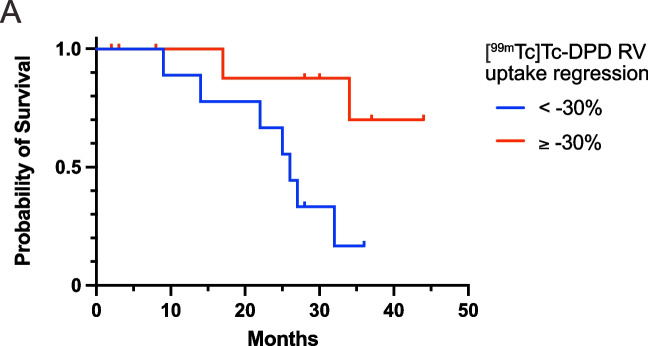
Kaplan–Meier Survival Curves: (**A**) Correlation between RV tracer regression (≥ −30% vs. < −30%) and long-term survival (log-rank *p* = 0.021)

In the Kaplan–Meier analysis, a longitudinal decrease in right ventricular [^99m^Tc]Tc-DPD tracer uptake equal to or greater than the median value at 12 month (−30%) was associated with a significant better survival (log-rank *p* = 0.021) (Fig. 4). The median longitudinal reduction in left ventricular [^99m^Tc]Tc-DPD tracer uptake at 12 month (−26%) did not provide significant prognostic information (log-rank *p* = 0.807).

In the univariable Cox proportional hazards regression analysis, a decrease in right ventricular [^99m^Tc]Tc-DPD tracer uptake equal to or greater than 30% at 12 month was associated with an improved survival (hazard ratio 0.18, 95% confidence interval 0.035–0.903, *p* = 0.037). A right ventricular [^99m^Tc]Tc-DPD tracer regression was still associated with reduced all-cause mortality when adjusted for clinical (NYHA functional class) and laboratory parameters (NT-proBNP, hs-cTnT indicated as Mayo score [1: NT-proBNP ≤ 3000 ng/L and hs-cTnT ≤ 50 ng/L, 2: NT-proBNP > 3000 ng/L or hs-cTnT > 50 ng/L, 3: NT-proBNP > 3000 ng/L and hs-cTnT > 50 ng/L]) in multivariable Cox regression analysis (hazard ratio 0.18, 95% confidence interval 0.034–0.951, *p* = 0.044). A detailed analysis can be found in the supplement (Table S1 in the Supplementary Appendix).

The median longitudinal reduction in left ventricular [^99m^Tc]Tc-DPD tracer uptake at 12 month (−26%) did not provide significant prognostic information in the Cox proportional hazards regression analysis (*p* = 0.807 and *p* = 0.387 for univariable and multivariable testing).

## Discussion

This single-center study is the first to investigate the effects of 6 and 12 months of tafamidis treatment on cardiac [^99m^Tc]Tc-DPD uptake in ATTR-CM. In parallel, the treatment effects on clinical parameters, cardiac biomarkers, and outcome were analyzed.

The key findings of our study were that treatment with tafamidis showed:

(i) A significant reduction in planar (Perugini score) and (semi-)quantitative cardiac tracer uptake at 6 and 12 months.
(ii) A significant improvement in clinical status and a notable decrease in NT-proBNP levels beginning already at 6 months and continuing at 12 months.
(iii) An association between the longitudinal reduction in quantitative RV tracer uptake at 12 months and improved patient long-term survival.

[^99m^Tc]Tc-DPD scintigraphy yields exceptional diagnostic accuracy in the recognition of ATTR-CM [9]. However, the clinical and prognostic relevance of serial [^99m^Tc]Tc-DPD scintigraphy in ATTR-CM remained unclear. In our study, significant declines in the Perugini scoring on planar imaging were documented at 6 and 12 months, which were mirrored by (semi-)quantitative tracer regression on SPECT/CT in tafamidis-treated ATTRwt-CM patients. This was paralleled by marked clinical improvements and a decrease in cardiac biomarkers.

A recent clinical follow-up study involving 40 patients with ATTRwt-CM, who underwent [^99m^Tc]Tc-DPD imaging at baseline and after 9 months of tafamidis treatment, recorded marked reductions in quantitative [^99m^Tc]Tc-DPD uptake. These were accompanied by clinical improvements, proposing serial scintigraphic imaging to be a valuable tool for monitoring treatment response [17]. The same study group also investigated the impact of the small interfering RNA patisiran on scintigraphic tracer uptake in ATTRv-CM and found similar reductions in quantitative myocardial uptake compared to the tafamidis treated ATTRwt-CM cohort [18]. Recently, data were reported from the NEURO-TTRansform trial, which included patients with ATTRv-CM receiving eplontersen. In a placebo-controlled setting, treatment with eplontersen led to a significant reduction in semiquantitative [^99m^Tc]Tc-Pyrophosphat (PYP) uptake. The study authors highlighted the potential utility of [^99m^Tc]Tc-PYP SPECT/CT for monitoring treatment efficacy and non-invasively assessing cardiac amyloid burden [21].

The exact mechanism behind the binding of [^99m^Tc]-labeled bone tracers to ATTR in the heart remains unclear. Therefore, the mechanism behind the reduction in cardiac tracer uptake over time with disease-specific therapy remains speculative. One possible explanation is that the treatment-induced inhibition of the ATTR tetramer dissociation leads to an actual reduction in myocardial amyloid load. This hypothesis is supported by the fact that there is a positive correlation between histologic amyloid load in endomyocardial biopsies and cardiac [^99m^Tc]Tc-DPD uptake as we have recently shown [15]. This finding was corroborated by another study demonstrating an association between cardiac [^99m^Tc]Tc-PYP uptake and histological amyloid load, suggesting that [^99m^Tc]-labeled bone tracers may indeed reflect true amyloid burden in ATTR-CM [16]. However, due to different dynamics and kinetics of [^99m^Tc]Tc-DPD binding to bones, skeletal muscles, and hearts of ATTR-CM patients caution was previously advised when interpreting changes in cardiac tracer uptake, particularly with [^99m^Tc]Tc-DPD. Changes in uptake in any of these three compartments may affect the calculation of cardiac tracer uptake [12]. Soft tissue uptake, primarily in planar imaging can confound results but can be overcome with additional SPECT/CT [22]. SPECT/CT is therefore crucial for identification of true cardiac tracer uptake. Another hypothesis suggests that disease-modifying therapy induces the replacement of amyloid deposits by fibrosis. This could explain the discrepancy between the lack of structural improvements in TTE, as shown in our study, and in CMR, as shown by others, but reduced tracer uptake on [^99m^Tc]Tc-DPD scintigraphy [6, 7]. This inconsistency emphasizes the need for future studies to establish the extent to which [^99m^Tc]Tc-DPD uptake reflects myocardial amyloid burden.

An association between baseline [^99m^Tc]-labeled bone scintigraphy and outcome has been suggested in previous studies [13, 14, 23]. We found a significant correlation between [^99m^Tc]Tc-DPD uptake regression at 12 months and patient outcome. In particular, regression in right ventricular [^99m^Tc]Tc-DPD uptake at 12 months was associated with improved long-term survival. However, considering the small sample size of our study cohort, further studies are needed to reevaluate the prognostic relevance of longitudinal tracer regression.

## Limitations

There are several limitations to be acknowledged in the present study. The data were collected in a single-center and were predominantly retrospective. Some ATTRwt-CM patients underwent [^99m^Tc]Tc-DPD scintigraphy solely at 6 months, while others underwent imaging only at 12 months. However, this is the first study to systematically conduct repetitive [^99m^Tc]Tc-DPD scintigraphy alongside clinical, echocardiographic and outcome assessments at various time points. The small sample size of our cohort significantly limits the statistical power regarding the correlation between survival and longitudinal [^99m^Tc]Tc-DPD uptake regression. Consequently, definitive conclusions regarding prognosis cannot be drawn. Further, our conclusions are limited to patients with wild-type ATTR-CM.

## Conclusions

In ATTRwt-CM patients, treatment with tafamidis was associated with marked reductions of [^99m^Tc]Tc-DPD tracer uptake on planar- and SPECT/CT imaging at 6 and 12 months. This was mirrored by clinical improvements and a decrease in cardiac biomarkers at 6 and 12 months of therapy. We also observed a significant correlation between the regression of right ventricular [^99m^Tc]Tc-DPD uptake at 12 months compared to baseline and long-term survival. Further studies are needed to generalize these findings.

## Supplementary Information

Below is the link to the electronic supplementary material.

Supplementary file1 (DOCX 21 KB)

## Data Availability

All data generated or analyzed during this study are included in this published article.

## Abbreviations

6MWT: 6-Minute walk test
[^99m^Tc]: Technetium-99 m
ATTR-CM: Transthyretin amyloid cardiomyopathy
ATTRv-CM: Variant transthyretin amyloid cardiomyopathy
ATTRwt-CM: Wild-type transthyretin amyloid cardiomyopathy
CMR: Cardiac magnetic resonance imaging
CPS: Counts of emitted radioactivity per pixel per second
DPD: 3,3-Diphosphono-1,2-propanodicarboxylic-acid
eGFR: Estimated glomerular filtration rate
GLS: Global longitudinal strain
H/CS: Heart-to-corpus-sterni ratio
H/M: Heart-to-mediastinum ratio
Hs-cTnT: High sensitivity cardiac troponin T
IVS: Interventricular septal thickness
LVEF: Left ventricular ejection fraction
NT-proBNP: N-terminal prohormone of brain natriuretic peptide
NYHA: New York Heart Association
PYP: Pyrophosphat
ROI: Region of interest
SPECT/CT: Single-photon emission computed tomography/computed tomography imaging
TTE: Transthoracic echocardiography
