# Supplementary material for: Effects of tafamidis on serial [99mTc]Tc-DPD scintigraphy in transthyretin amyloid cardiomyopathy
Source: Eur J Nucl Med Mol Imaging. 2025 Feb 6;52(7):2529–37. doi: 10.1007/s00259-025-07092-7 (PMC12119690; doi:10.1007/s00259-025-07092-7)
Supplement: Supplementary file 1 — Supplementary file1 (DOCX 21 KB) [file 259_2025_7092_MOESM1_ESM.docx]

Supplementary Appendix

Supplementary Table S1. Multivariable analysis: All-cause mortality

| Variables | Hazard Ratio [95 % CI] | p-value |
| --- | --- | --- |
| RV tracer regression, ≥ -30 %^a^ | 0.18 [0.034-0.951] | **0.044** |
| NYHA, II to III class increase^b^ | 4.51 [1.022-19.890] | **0.047** |
| Mayo score, per 1 score increase | 1.47 [0.466-4.622] | 0.512 |

Values are Hazard Ratio [95 % Confidence Interval].

^a^Median right ventricular [^99m^Tc]Tc-DPD tracer regression from baseline to 12 months.

^b^No patients with NYHA functional class I or IV were available for RV tracer regression analysis.
